# Supplementary material for: Integrating Multiple Genomic Data to Predict Disease-Causing Nonsynonymous Single Nucleotide Variants in Exome Sequencing Studies
Source: PLoS Genet. 2014 Mar 20;10(3):e1004237. doi: 10.1371/journal.pgen.1004237 (PMC3961190; doi:10.1371/journal.pgen.1004237)
Supplement: Text S1 — Supplementary results. (PDF) [file pgen.1004237.s004.pdf]

Supplementary Results for

Integrating Multiple Genomic Data to Predict Disease-causing Nonsynonymous Single Nucleotide Variants in Exome Sequencing Studies

Jiaxin Wu [1], Yanda Li [1], Rui Jiang [1 §]

[1] MOE Key Laboratory of Bioinformatics; Bioinformatics Division and Center for Synthetic & Systems Biology, TNLIST; Department of Automation, Tsinghua University, Beijing, China

[§] Corresponding author. Email: ruijiang@tsinghua.edu.cn

Description of the exome sequencing data for simulation studies

We downloaded exome sequencing data of eight HapMap individuals that represented three populations (Europe, Asia and Africa) and derived a set of nonsynonymous SNVs for each individual (Supplementary Table 1). We checked overlaps of these sets of SNVs (Supplementary Table 2). Briefly, there are a total of 23,811 SNVs existing in at least one exome, and 21,947 of them can be mapped to either dbNSFP or the genomic data sources. Among the 21,947 SNVs, 10,739 (48.93%) are uniquely mapped to only one exome, 3,266 (14.88%) mapped to two exomes, and only a small number of 1,563 (7.12%) SNVs shared by all the eight exomes. We then calculated their pairwise Jaccard coefficients (Supplementary Table 3) and found that small Jaccard coefficients (< 0.5) were achieved between every two sets of SNVs, indicating that the SNVs derived from the eight exomes were significantly different from each other.

Statistical significance of candidate SNVs in validation experiments

We analyzed the statistical significance of candidate SNVs in validation experiments for diseases with partly known genetic bases (Supplementary Figure 1, A and B). When compared with the neutral control set, the median q-values of the test and control SNVs are 6.70×10^{-7} and 0.8921, respectively. When compared with the disease control set, the median q-values of the test and control SNVs are 3.00×10^{-8} and 1.50×10^{-3}, respectively. When compared with the combined control set, the median q-values of the test and control SNVs are 6.00×10^{-8} and 0.0291, respectively. These results indicate that the q-values of test SNVs are typically much smaller than those of control ones. Hence, when ranking a test SNV against control ones according to their q-values, the test SNV is likely to be ranked among top positions.

We also analyzed the statistical significance of candidate SNVs in validation experiments for diseases of unknown genetic bases (Supplementary Figure 1, C and D). When compared with the neutral control set, the median q-values of the test and control SNVs are 2.51×10^{-3} and 0.9998, respectively. When compared with the disease control set, the median q-values of the test and control SNVs are 1.06×10^{-4} and 0.0295, respectively. When compared with the combined control set, the median q-values of the test and control SNVs are 1.93×10^{-4} and 0.2515, respectively. These results also indicate that the q-values of test SNVs are typically much smaller than those of control ones.

We notice that median q-values of test SNVs for diseases with partly known genetic bases are typically smaller than those for diseases with unknown genetic bases

(Supplementary Figure 1, A versus C). This is understandable since seed genes selected for the later, as a collection of genes known as associated with 10 diseases of the highest phenotype similarities to the query disease, might not be as reliable as those for the former.

Effects of the number of seed genes and the number of neighbouring diseases

The number of known disease-causing genes is quite different for different diseases whose genetic bases are partly known. On this scenario, we assessed whether the number of seed genes affect the prediction power of SPRING in detecting causative SNVs. We divided the 113 diseases annotated as associated with at least two genes into four groups: the first group contained 71 diseases with 2 seed genes; the second group contained 20 diseases with 3 seed genes; the third group contained 11 diseases with 4 seed genes; the fourth group included 11 diseases with the number of seed genes ranging from 5 to 12. We then repeated the validation experiment for each group. Results, as summarized in Supplementary Figure 2, demonstrate that the number of known disease-causing genes has little influence on the effectiveness of SPRING for partly known disease detection, since both the MRRs and the AUCs only show reasonable fluctuation among these groups.

In the detection of causative SNVs for query diseases whose genetic bases were unknown, we selected seed genes for a query disease as genes annotated as associated with 10 diseases of the highest phenotype similarities with the query disease. We then assessed how the number of such neighboring diseases affected the prediction power of our method by evaluating the performance of SPRING for all the 1,436 diseases with seed genes selected as those associated with 2, 4, 6, 8, 10, 12, and 15 diseases of the highest phenotype similarities with the query disease. Results, as shown in Supplementary Figure 3, illustrate the robustness of our method to the number of neighboring diseases. Briefly, when the number of neighboring diseases is smaller than or equal to 6, the performance of our method exhibits improvement with the increase of the neighbouring diseases. When the number of neighboring diseases is greater than or equal to 8, the performance of our method is quite stable, only showing slight fluctuations with different numbers of neighbouring diseases.

We further explored the reason behind the above observations. Typically, diseases share common causative genes have high phenotype similarity (Wu et al. 2009). Therefore, as the number of neighboring diseases increases, genes causative for a query disease will likely be included in the seed genes for the disease, and hence the performance of our method show improvements. On the opposite side, when the number of neighboring disease is too large,

some redundant, useless or even wrong information about seed genes may be included, and thus the performance of our method on some diseases decreases, preventing further improvement of the overall performance. Moreover, it is obvious that the computational burden will increase as the number of neighboring disease increases. Therefore, to achieve a reasonable balance between the effectiveness and efficiency of our method, we select 10 neighboring diseases by default.

Functional effect scores of rare SNVs

We compared distributions of functional effect scores for these rare SNVs against those for the same number of SNVs selected at random from one of the eight HapMap individuals. Results show that the rare SNVs are typically assigned more extreme functional effect scores than SNVs occurring in a normal individual (Supplementary Figure 4). Since the sequence conservation property has been used in the derivation of the functional effect scores, we conjecture that the effectiveness of our method in this validation experiment can be partly attributed to the rarity of such rare mutations in a random human.

Distributions of association scores for neutral and disease controls

For each of the 1,436 diseases in the validation experiments for diseases of unknown genetic bases, we calculated association scores for neutral and disease controls for each of the five genomic data sources. We then selected for each data source 100,000 scores at random from the resulting scores to draw distributions of association scores for neutral and disease controls. Results show that the distributions for these two groups of variants are not significantly different (Supplementary Figure 5). This observation helps to explain the reason why an association data source exhibits comparable effectiveness in distinguishing disease-causing SNVs from neutral, disease, and combined controls.

Selection of data sources for individual diseases

We calculated the pairwise Pearson's correlation coefficient of the data sources. Results show that the data sources are correlated (Supplementary Figure 6). We further adopted a sequential backward selection (SBS) strategy, a greedy algorithm, to select a subset of data sources for each of the 1,436 diseases. Briefly, for a certain disease, we started from a set including all data sources, repeatedly removed the data source of the least importance with respect to the remaining data sources until the smallest MRR was achieved, and obtained a subset of selected data sources as the retained ones. The results are summarized in Supplementary Table 4.

First, we observe that the coverage of functional effect data sources for different diseases is almost the same. For association data sources, the coverage of pathway and domain data is lower than that of PPI, whose coverage is in turn lower than that of GO and sequence data. Moreover, two-sided Fisher's exact tests with multiple testing corrections do not support the existence of biases on the presences of individual data sources in Mendelian and complex diseases. A similar statistical comparison also suggests that the presences of individual data sources in immune and neurological disorders are not significantly different.

Second, we notice that GO, PPI, and pathway data are selected by more than 70% diseases (with the consideration of the presence of a data source before the selection procedure), while all the other data sources are selected by about half of the diseases, suggesting all data sources are important to our method. For Mendelian diseases, the pattern of selection is similar to those for all diseases. For complex diseases, PPI and pathway data are selected by a larger proportion of diseases, suggesting these data may play more important roles for these diseases. However, two-sided Fisher's exact tests with multiple testing corrections do not support the existence of significant difference between Mendelian and complex diseases in the selection of individual data sources. A similar statistical comparison also suggests that the selection of individual data sources is not significantly different between immune diseases and neurological disorders.

Third, we notice that the selection procedure is helpful in improving the performance of our method. For example, using only selected data sources, the average MRR for all the 1,436 diseases improves from 0.1265 to 0.0791, and the average AUC increases from 0.8735 to 0.9209 (Table S2).

Finally, we notice that the selected subsets of data sources are preferred by quite different numbers of diseases (Table S2). There are a total of 308 distinct subsets of data sources selected by the 1,436 diseases. Among these subsets, 273 diseases favor the use of all the 11 data sources, 175 diseases preferring all but the pathway information, and 82 diseases preferring all but the domain information. The total number of diseases preferring the most selected 21 subsets is 911, while the rest 287 subsets are only selected by 525 diseases. When looking deeply at the improvement in the performance of our method with respect to the number of selected data sources (Supplementary Figure 7), we notice that our method is more effective for diseases preferring more data sources, since the performance of our method for a total of 627 (43.66%) diseases that prefer 6 or more data sources is typically higher than that of 809 (56.34%) diseases that prefer 5 or less data sources. For diseases preferring more data

sources, the improvement after selection is marginal. For diseases favoring less data sources, the performance does exhibit significant improvement with the selection procedure. Nevertheless, such diseases exhibit quite different preferences. For example, there are a total of 275 distinct subsets of data sources selected by diseases that prefer 5 or less data sources, and on average each subset is only selected by 809/275 ≈ 2.94 diseases (Table S2).

From these results, we make the following conclusions. First, the procedure of selecting of a subset of data sources can improve the performance of our approach, especially for more than half diseases that prefer less data sources. Second, the selection procedure should be done for individual diseases, since different diseases show different preferences on the selected data sources and such preferences are diverse. Third, considering that the selection procedure has marginal effects on about 43.66% diseases in our experiment, and for the rest of 56.34% diseases the preference is diverse, we suggest either seeking for the simplicity to use all data sources without selection or resorting to a cross-validation experiment to select a subset of data sources for a query disease when there is no strong prior knowledge indicating the preference of the disease to the data sources. In this paper, we use all data sources without selection by default unless otherwise stated.

Estimation of the false positive rate and true positive rate

To estimate the false positive rate (FPR) and the true positive rate (TPR) at a given q-value cut-off, we generated at random 12 sets of SNVs, each composed of 10,000 SNVs. In each set, the proportion of disease SNVs ranges from 0% to 100% separately. We then calculated q-values for SNVs in every set, estimated the false positive rate as the proportion of neutral SNVs whose q-values are less than or equal to the threshold and the true positive rate at a certain q-value threshold as the proportion of disease SNVs whose q-values are less than or equal to the threshold. The results of the false positive rates and the true positive rates at different q-value cut-offs are summarized in Supplementary Figure 8.

When the SNV set contains equal number of neutral SNVs and disease SNVs, the estimated false positive rates at the q-value threshold 0.1, 0.05, 0.01 and 0.005 are 12.93%, 9.37%, 4.23% and 3.08% respectively, and the estimated true positive rates are 93.56%, 90.62%, 82.84% and 79.32% respectively. Compared with SIFT (Kumar et al. 2009) (FPR=20.0%, TPR=69.0%), PolyPhen2 (Adzhubei et al. 2010) (FPR=21.1%, TPR=77.3%), and MutationTaster (Schwarz et al. 2010) (FPR=14.5%, TPR=85.9%), our method clearly achieves significantly lower false positive rate and higher true positive rate than these

methods in the prediction of causative SNVs. When the SNV set contains a small fraction of disease SNVs, say 5%, the estimated false positive rates at the *q*-value threshold 0.1, 0.05, 0.01 and 0.005 are 6.38%, 4.08%, 1.69% and 0.56% respectively, and the estimated true positive rates are 86.28%, 82.08%, 72.42% and 68.46% respectively. When the SNV set contains no disease SNVs, the estimated false positive rates at the *q*-value threshold 0.1, 0.05, 0.01 and 0.005 are 4.18%, 2.43%, 0.71% and 0.48% respectively.

Detection of causative nonsynonymous *de novo* mutations for autism, epileptic encephalopathies and intellectual disability

Recent studies based on whole-exome sequencing have shown that highly disruptive nonsense and splice site *de novo* mutations in brain-expressed genes exhibit strong associations with autism, revealing potential large impacts of *de novo* mutations on the pathogenesis of this disease (Iossifov et al. 2012; Neale et al. 2012; O'Roak et al. 2012; Sanders et al. 2012). Besides the application of our method to PMID 22495306 (Sanders et al. 2012) as described in the main text, we further applied SPRING to three independent exome sequencing data sets for autism, including PMID 22495309 (O'Roak et al. 2012), PMID 22495311 (Neale et al. 2012), and PMID 22542183 (Iossifov et al. 2012).

PMID 22495309

From the literature (O'Roak et al. 2012), we collected 160 unique candidate nonsynonymous *de novo* mutations from the exome sequencing data of 189 probands, and 154 of these mutations can be mapped to the dbNSFP database. With the criterion that a seed gene should have been reported as associated with autism by independent studies before the publication of this data set, we selected from the OMIM database 34 seed genes and then applied SPRING to prioritize the candidate mutations (Table S3).

In the literature (O'Roak et al. 2012), 41 mutations were reported as contributing to the risk of autism, based on functional damaging scores, functional evidence, or previous studies. SPRING successfully ranked 8 of these mutations among top 10 and 14 among top 20. At the *q*-value cut-off 0.01, 26 mutations were reported by SPRING, and 17 of them were claimed as risk contributing according to the literature (O'Roak et al. 2012). Using Fisher’s exact test, we estimated that the probability of ranking 8 or more mutations among top 10 by change was 4.21×10^{-4}, and that of ranking 14 or more mutations among top 20 by change was 1.55×10^{-5}, both supporting the effectiveness of our method. We also adopted the one-sided Wilcoxon

rank sum test to check whether the q-values of the reported 41 disease-associated mutations were significantly less than those of the other candidate mutations (suppose irrelevant to autism) and obtained a p-value of 8.03×10^{-9}.

We noticed that CHD8 (Neale et al. 2012), NTNG1 (O'Roak et al. 2011), PTEN (Butler et al. 2005) and TBL1XR1 (Chung et al. 2011) had been previously annotated as causative for autism by independent studies and thus was included in seed genes in the above analysis. To simulate the scenario of identifying causative variants occurring in genes not yet studied, we removed CHD8, NTNG1, PTEN and TBL1XR1 from the seed genes and prioritized the candidate mutations again. We found that SPRING ranked 7 of the reported causative mutations among top 10 and 13 among top 20. Using Fisher's exact test, we estimated that the probability of ranking 7 or more mutations among top 10 by change was 3.85×10^{-3}, and that of ranking 13 or more mutations among top 20 by change was 1.22×10^{-4}. The one-sided Wilcoxon rank sum test yielded a p-value of 1.10×10^{-7}, suggesting the q-values of the reported 41 disease-associated mutations were significantly less than those of the other candidate mutations.

PMID 22495311

From the literature (Neale et al. 2012), we collected 111 unique candidate nonsynonymous *de novo* mutations from the exome sequencing data of 175 probands, and 107 of these mutations can be mapped to the dbNSFP database. With the criterion that a seed gene should have been reported as associated with autism by independent studies before the publication of this data set, we selected the 34 seed genes identified previously and then applied SPRING to prioritize the candidate mutations (Table S3).

In the literature (Neale et al. 2012), 5 mutations were reported as causative for autism, and none of them occurred in genes previously annotated as causative for this disease. SPRING ranked these mutations at 2, 3, 7, 10 (tie with 3 other mutations), and 52. At the q-value cut-off 0.01, SPRING reported 13 mutations, among which 4 were reported in the literature (Neale et al. 2012). Using Fisher's exact test, we estimated that the probability of ranking 3 or more mutations among top 10 by change was 5.45×10^{-3}, and that of ranking 4 or more mutations among top 20 by change was 4.11×10^{-3}, both supporting the effectiveness of our method. We also adopted the one-sided Wilcoxon rank sum test to check whether the q-values of the reported 5 disease-associated mutations were significantly less than those of the other candidate mutations and obtained a p-value of 2.19×10^{-3}.

PMID 22542183

From the literature (Iossifov et al. 2012), we collected 1,528 unique candidate nonsynonymous *de novo* mutations from the exome sequencing data of 343 families, and all these mutations can be mapped to the dbNSFP database. With the criterion that a seed gene should have been reported as associated with autism by independent studies before the publication of this data set, we selected the 34 seed genes identified previously and then applied SPRING to prioritize the candidate mutations (Table S3).

In the literature (Iossifov et al. 2012), 18 mutations were reported “likely gene disrupting” (LGD) in affected children and 116 were reported belonging to FMR1 targets. SPRING reported 4 such mutations among top 10 and 6 among top 20. At the *q*-value cut-off 0.01, SPRING reported 43 mutations, among which 13 were reported either LGD or belonging to the FMR1 targets according to the literature (Iossifov et al. 2012). Using Fisher’s exact test, we estimated that the probability of ranking 4 or more mutations among top 10 by change was only 7.82×10^{-3}, and that of ranking 6 or more mutations among top 20 by change was only 5.62×10^{-3}, both supporting the effectiveness of our method. We also adopted the one-sided Wilcoxon rank sum test to check whether the *q*-values of the reported 134 disease-associated mutations were significantly less than those of the other candidate mutations and obtained a *p*-value smaller than 2.2×10^{-16}.

We noticed that SCN2A (Weiss et al. 2003) had been previously annotated as causative for autism by an independent study and thus was included in seed genes in the above analysis. To simulate the scenario of identifying causative variants occurring in genes not yet studied, we removed SCN2A from the seed genes and prioritized the candidate mutations again. We found that SPRING ranked 4 of these mutations among top 10 and 8 among top 20. Using Fisher’s exact test, we estimated that the probability of ranking 4 or more mutations among top 10 by change was 7.82×10^{-3}, and that of ranking 8 or more mutations among top 20 by change was 1.45×10^{-4}. The one-sided Wilcoxon rank sum test yielded a *p*-value smaller than 2.2×10^{-16}, suggesting the *q*-values of the reported 134 disease-associated mutations were significantly less than those of the other candidate mutations.

In a recent study (Allen et al. 2013), exome sequencing was successfully applied to the detection of causal genetic variants for epileptic encephalopathies, showing strong statistical evidence on the association of several *de novo* mutations with this group of complex diseases. We therefore applied SPRING to the analysis of exome sequencing data in this study (PMID 23934111 (Allen et al. 2013)).

PMID 23934111

From the literature (Allen et al. 2013), we collected 192 unique candidate nonsynonymous *de novo* mutations from the exome sequencing data of 264 probands and their parents, and all these mutations can be mapped to the dbNSFP database. With the criterion that a seed gene should have been reported as associated with epileptic encephalopathies by independent studies before the publication of this data set, we selected from the OMIM database a total of 15 seed genes and then applied SPRING to prioritize the candidate mutations (Table S3).

In the literature (Allen et al. 2013), the authors discovered 30 likely functional *de novo* mutations. SPRING successfully ranked 10 of these mutations among top 10 and 17 among top 20. At the *q*-value cut-off 0.01, SPRING reported 18 mutations, among which 17 had been reported as likely functional (Allen et al. 2013). Using Fisher's exact test, we estimated that the probability of ranking 10 or more mutations among top 10 by change was only 2.03×10^{-9}, and that of ranking 17 or more mutations among top 20 by change was only 1.24×10^{-13}, both strongly supporting the effectiveness of our method. We also adopted the one-sided Wilcoxon rank sum test to check whether the *q*-values of the reported 30 disease-associated mutations were significantly less than those of the other candidate mutations (suppose irrelevant to epileptic encephalopathies) and obtained a *p*-value of 1.19×10^{-11}.

We noticed that CDKL5 (Rosas-Vargas et al. 2008), KCNQ2 (Borgatti et al. 2004), SCN1A (Ohmori et al. 2002), SCN2A (Meisler and Kearney 2005), SCN8A (Veeramah et al. 2012) and STXBP1 (Saitsu et al. 2011) had been previously annotated as causative for epileptic encephalopathies by an independent study and thus was included in seed genes in the above analysis. To simulate the scenario of identifying causative variants occurring in genes not yet studied, we removed CDKL5, KCNQ2, SCN1A, SCN2A, SCN8A and STXBP1 from the seed genes and prioritized the candidate mutations again. We found that SPRING ranked 4 of these mutations among top 10 and 9 among top 20. Using Fisher's exact test, we estimated that the probability of ranking 4 or more likely functional mutations among top 10 by change was 5.20×10^{-2}, and that of ranking 9 or more mutations among top 20 by change was 8.76×10^{-4}. The one-sided Wilcoxon rank sum test yielded a *p*-value of 4.89×10^{-8}, suggesting the *q*-values of the reported 30 disease-associated mutations were significantly less than those of the other candidate mutations.

We further extended the application of SPRING to the analysis of two independent data sets of intellectual disability (PMID 23033978 (Neale et al. 2012) and PMID 23020937 (Iossifov et al. 2012)).

PMID 23033978

From the literature (Neale et al. 2012), we collected 78 unique candidate nonsynonymous *de novo* mutations from the exome sequencing data of 53 patients, and 77 of these mutations can be mapped to the dbNSFP database. Since there is no exact entry for intellectual disability in the OMIM database, we selected the 15 genes associated with epileptic encephalopathies as seeds (Table S3).

In the literature (Neale et al. 2012), the authors discovered 16 risk contributing *de novo* mutations for intellectual disability. When looking at the ranking list produced by SPRING, we found that 3 mutations ranked among top 10 and 8 among top 20 were reported as associated with intellectual disability. At the *q*-value cut-off 0.1, SPRING reported 30 mutations, among which 10 had been reported as associated with intellectual disability according to the literature (Neale et al. 2012). Using Fisher's exact test, we estimated that the probability of ranking 3 or more mutations among top 10 by change was 0.34, and that of ranking 8 or more mutations among top 20 by change was 1.90×10^{-2}. We also adopted the one-sided Wilcoxon rank sum test to check whether the *q*-values of the reported 16 risk contributing mutations were significantly less than those of the other candidate mutations and obtained a *p*-value of 3.02×10^{-2}.

We noticed that SCN2A (Meisler and Kearney 2005) had been previously annotated as causative for intellectual disability by an independent study and thus was included in seed genes in the above analysis. To simulate the scenario of identifying causative variants occurring in genes not yet studied, we removed SCN2A from the seed genes and prioritized the candidate mutations again. We found that SPRING ranked 4 of these mutations among top 10 and 8 among top 20. Using Fisher's exact test, we estimated that the probability of ranking 4 or more mutations among top 10 by change was 0.12, and that of ranking 8 or more mutations among top 20 by change was 1.90×10^{-2}. The one-sided Wilcoxon rank sum test yielded a *p*-value of 3.57×10^{-2}, suggesting the *q*-values of the reported 16 disease-associated mutations were significantly less than those of the other candidate mutations.

PMID 23020937

From the literature (Iossifov et al. 2012), we collected 131 unique candidate nonsynonymous *de novo* mutations from the exome sequencing data of 51 patients, and 126 of these mutations can be mapped to the dbNSFP database. We also selected the 15 genes associated with epileptic encephalopathies as seeds (Table S3).

In the literature (Iossifov et al. 2012), the authors discovered 17 possibly disease-causing mutations. When looking at the ranking list produced by SPRING, we found that 6 mutations ranked among top 10 and 7 among top 20 were reported as associated with intellectual disability. At the *q*-value cut-off 0.01, SPRING reported 5 mutations, and all of them were reported as disease-causing in the literature (Iossifov et al. 2012). Using Fisher's exact test, we estimated that the probability of ranking 6 or more mutations among top 10 by change was 3.79×10^{-4}, and that of ranking 7 or more mutations among top 20 by change was 6.36×10^{-3}, both supporting the effectiveness of our method. We also adopted the one-sided Wilcoxon rank sum test to check whether the *q*-values of the reported 17 disease-associated mutations were significantly less than those of the other candidate mutations and obtained a *p*-value of 2.63×10^{-5}.

We noticed that SCN2A (Meisler and Kearney 2005), SCN8A (Veeramah et al. 2012) and STXBP1 (Saitsu et al. 2011) had been previously annotated as causative for intellectual disability by an independent study and thus was included in seed genes in the above analysis. To simulate the scenario of identifying causative variants occurring in genes not yet studied, we removed SCN2A, SCN8A and STXBP1 from the seed genes and prioritized the candidate mutations again. We found that SPRING ranked 4 of these mutations among top 10 and 6 among top 20. Using Fisher's exact test, we estimated that the probability of ranking 4 or more mutations among top 10 by change was 2.92×10^{-2}, and that of ranking 6 or more mutations among top 20 by change was 2.97×10^{-2}. The one-sided Wilcoxon rank sum test yielded a *p*-value of 6.02×10^{-5}, suggesting the *q*-values of the reported 17 disease-associated mutations were significantly less than those of the other candidate mutations.

These results confirm the capability of SPRING in identifying causative nonsynonymous *de novo* mutations for complex diseases, not only in the case of finding novel causative variants occurring in known causative genes, but also in the situation of detecting causative variants occurring in genes not yet studied.

References

Adzhubei IA, Schmidt S, Peshkin L, Ramensky VE, Gerasimova A et al. A method and server for predicting damaging missense mutations. *Nature methods*, **7**(4): 248-249, 2010.

Allen AS, Berkovic SF, Cossette P, Delanty N, Dlugos D et al. De novo mutations in epileptic encephalopathies. *Nature*, **501**(7466): 217-221, 2013.

Borgatti R, Zucca C, Cavallini A, Ferrario M, Panzeri C et al. A novel mutation in KCNQ2 associated with BFNC, drug resistant epilepsy, and mental retardation. *Neurology*, **63**(1): 57-65, 2004.

Butler M, Dasouki M, Zhou X, Talebizadeh Z, Brown M et al. Subset of individuals with autism spectrum disorders and extreme macrocephaly associated with germline PTEN tumour suppressor gene mutations. *Journal of medical genetics*, **42**(4): 318-321, 2005.

Chung R-H, Ma D, Wang K, Hedges DJ, Jaworski JM et al. An X chromosome-wide association study in autism families identifies TBL1X as a novel autism spectrum disorder candidate gene in males. *Mol Autism*, **2**(1): 18-18, 2011.

Iossifov I, Ronemus M, Levy D, Wang Z, Hakker I et al. De novo gene disruptions in children on the autistic spectrum. *Neuron*, **74**(2): 285-299, 2012.

Kumar P, Henikoff S, Ng PC. Predicting the effects of coding non-synonymous variants on protein function using the SIFT algorithm. *Nature protocols*, **4**(7): 1073-1081, 2009.

Meisler MH, Kearney JA. Sodium channel mutations in epilepsy and other neurological disorders. *Journal of Clinical Investigation*, **115**(8): 2010-2017, 2005.

Neale BM, Kou Y, Liu L, Ma'ayan A, Samocha KE et al. Patterns and rates of exonic de novo mutations in autism spectrum disorders. *Nature*, **485**(7397): 242-245, 2012.

O'Roak BJ, Deriziotis P, Lee C, Vives L, Schwartz JJ et al. Exome sequencing in sporadic autism spectrum disorders identifies severe de novo mutations. *Nature genetics*, **43**(6): 585-589, 2011.

O'Roak BJ, Vives L, Girirajan S, Karakoc E, Krumm N et al. Sporadic autism exomes reveal a highly interconnected protein network of de novo mutations. *Nature*, **485**(7397): 246-250, 2012.

Ohmori I, Ouchida M, Ohtsuka Y, Oka E, Shimizu K. Significant correlation of the SCN1A mutations and severe myoclonic epilepsy in infancy. *Biochemical and biophysical research communications*, **295**(1): 17-23, 2002.

Rosas-Vargas H, Bahi-Buisson N, Philippe C, Nectoux J, Girard B et al. Impairment of CDKL5 nuclear localisation as a cause for severe infantile encephalopathy. *Journal of medical genetics*, **45**(3): 172-178, 2008.

Saitsu H, Hoshino H, Kato M, Nishiyama K, Okada I et al. Paternal mosaicism of an STXBP1 mutation in OS. *Clinical genetics*, **80**(5): 484-488, 2011.

Sanders SJ, Murtha MT, Gupta AR, Murdoch JD, Raubeson MJ et al. De novo mutations revealed by whole-exome sequencing are strongly associated with autism. *Nature*, **485**(7397): 237-241, 2012.

Schwarz JM, Rödelsperger C, Schuelke M, Seelow D. MutationTaster evaluates disease-causing potential of sequence alterations. *Nature methods*, **7**(8): 575-576, 2010.

Veeramah KR, O'Brien JE, Meisler MH, Cheng X, Dib-Hajj SD et al. De Novo Pathogenic SCN8A Mutation Identified by Whole-Genome Sequencing of a Family Quartet Affected by Infantile Epileptic Encephalopathy and SUDEP. *The American Journal of Human Genetics*, **90**(3): 502-510, 2012.

Weiss LA, Escayg A, Kearney JA, Trudeau M, MacDonald BT et al. Sodium channels SCN1A, SCN2A and SCN3A in familial autism. *Molecular psychiatry*, **8**(2): 186-194, 2003.

Wu X, Liu Q, Jiang R. Align human interactome with phenome to identify causative genes and networks underlying disease families. *Bioinformatics*, **25**(1): 98-104, 2009.

Supplementary Figures

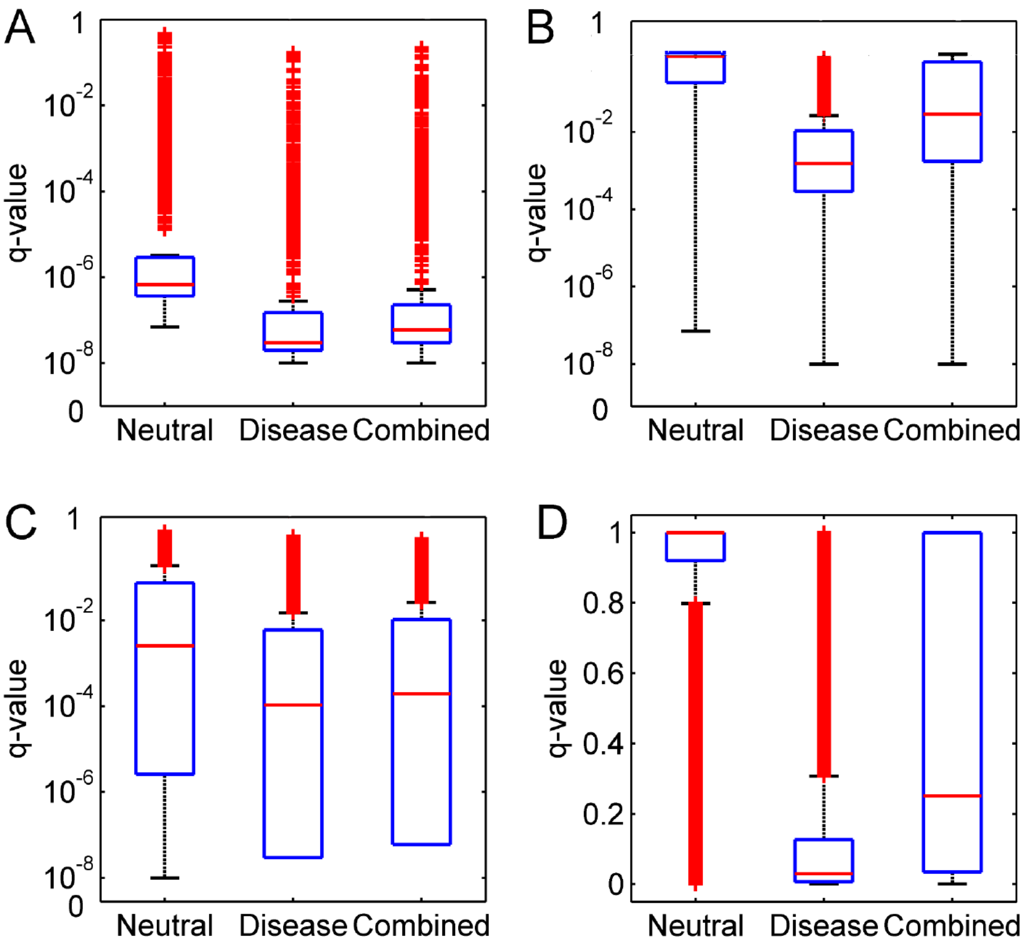

Supplementary Figure 1. Boxplots of *q*-values for (A) test SNVs for diseases with partly known genetic bases, (B) control SNVs for diseases with partly known genetic bases, (C) test SNVs for diseases of unknown genetic bases, and (D) control SNVs for diseases of unknown genetic bases.

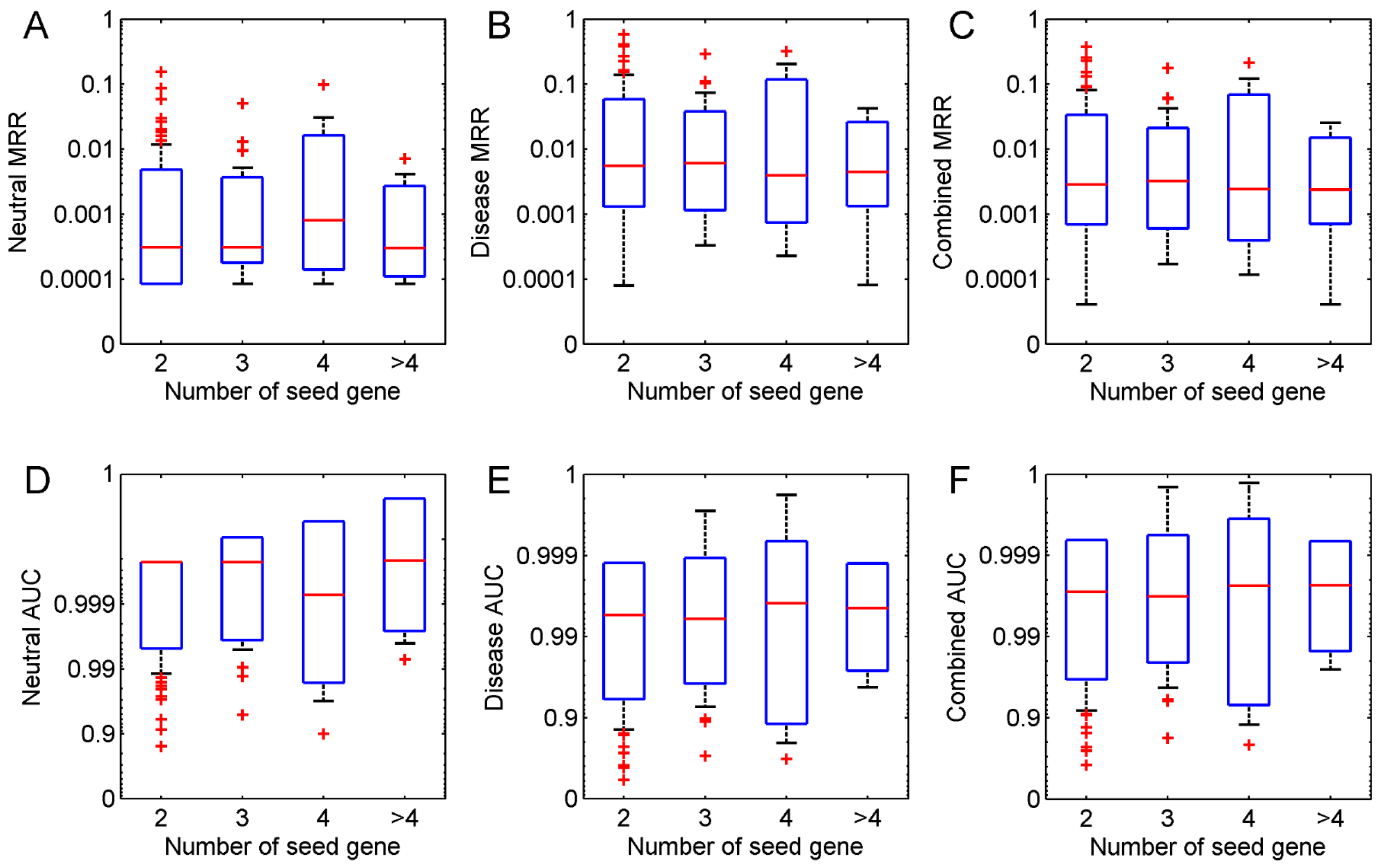

Supplementary Figure 2. Effects of the number of seed genes. (A) MRRs when validating against the neutral control set. (B) MRRs when validating against the disease control set. (C) MRRs when validating against the combined control set. (D) AUCs when validating against the neutral control set. (E) AUCs when validating against the disease control set. (F) AUCs when validating against the combined control set.

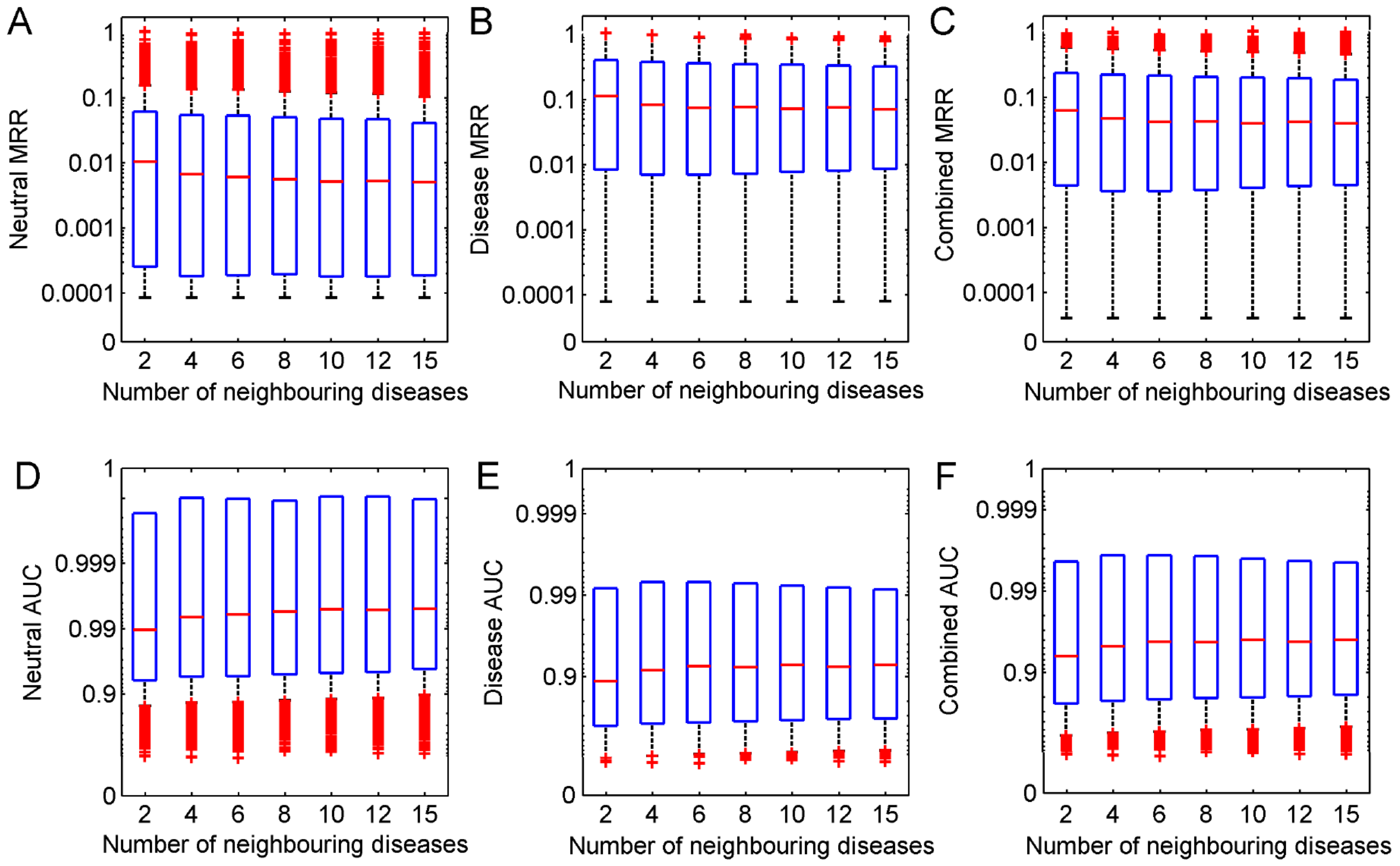

Supplementary Figure 3. Effects of the number of neighbouring diseases. (A) MRRs when validating against the neutral control set. (B) MRRs when validating against the disease control set. (C) MRRs when validating against the combined control set. (D) AUCs when validating against the neutral control set. (E) AUCs when validating against the disease control set. (F) AUCs when validating against the combined control set.

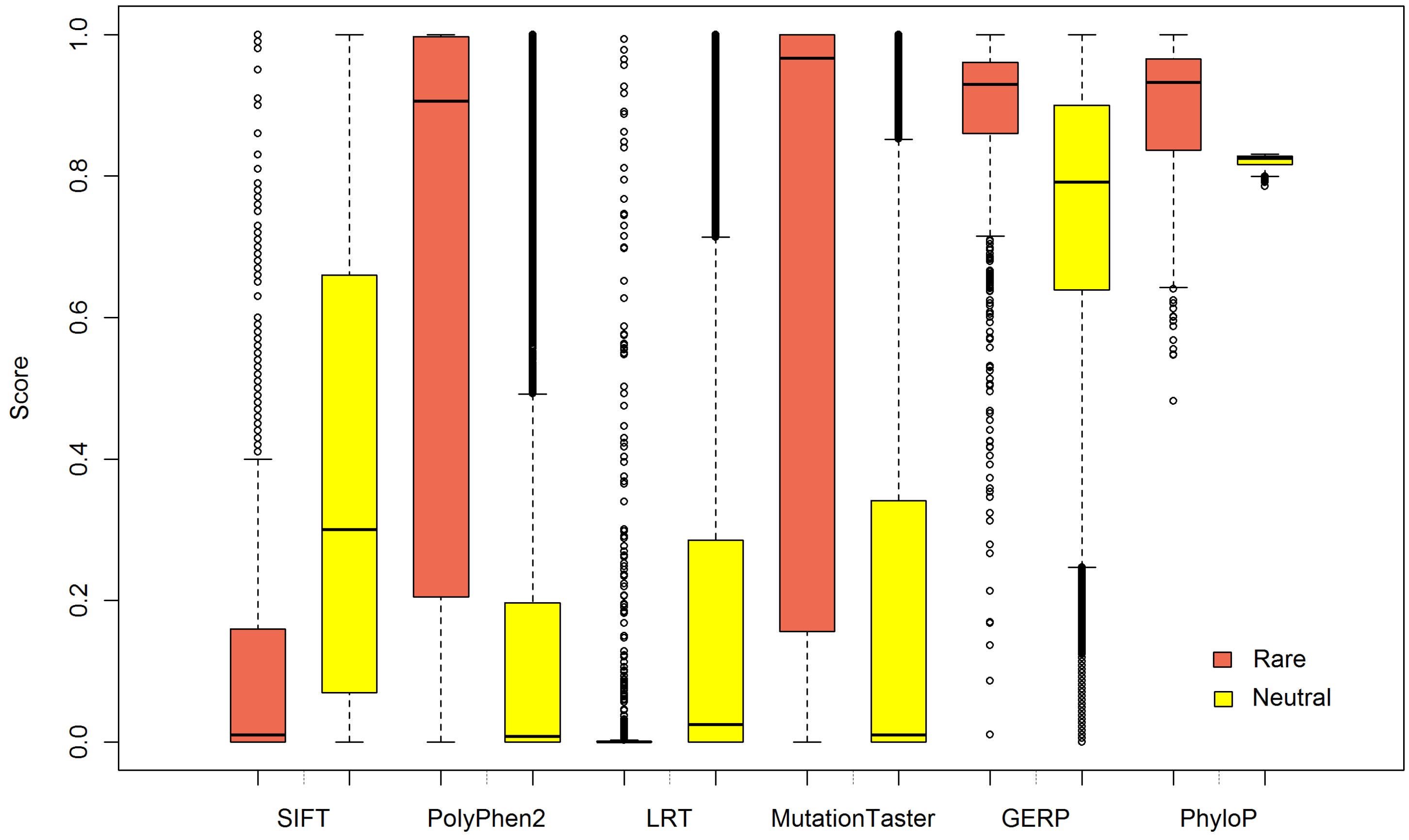

Supplementary Figure 4. Boxplots of functional effect scores for the rare SNVs. GERP and PhyloP scores have been normalized to the interval of [0,1] by a linear transformation.

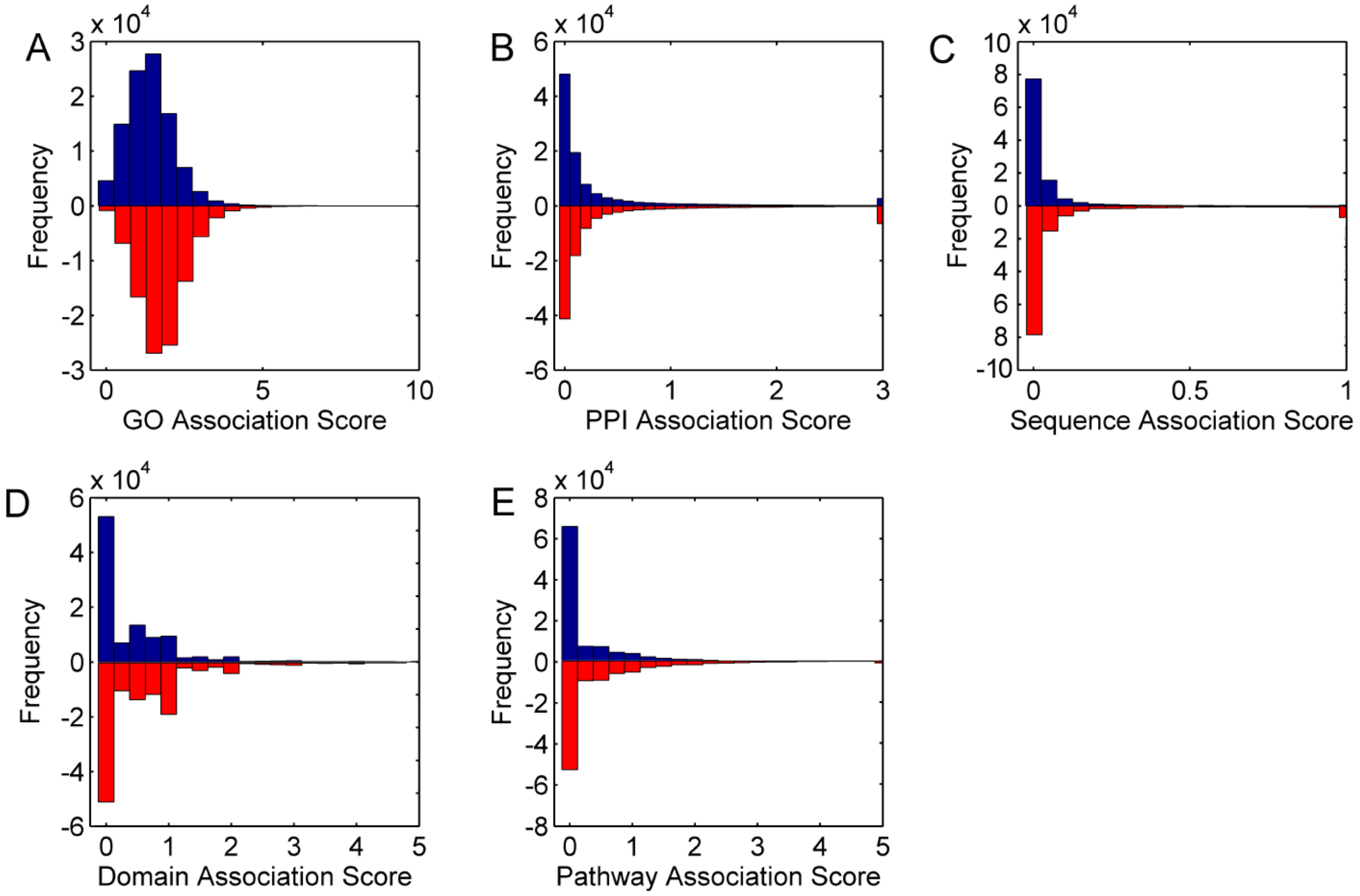

Supplementary Figure 5. Distributions of association scores of the neutral control set (blue) and the disease control set (red). (A) Association scores derived from GO. (B) Association scores derived from PPI. (C) Association scores derived from protein sequence. (D) Association scores derived from protein domain. (E) Association scores derived from pathway.

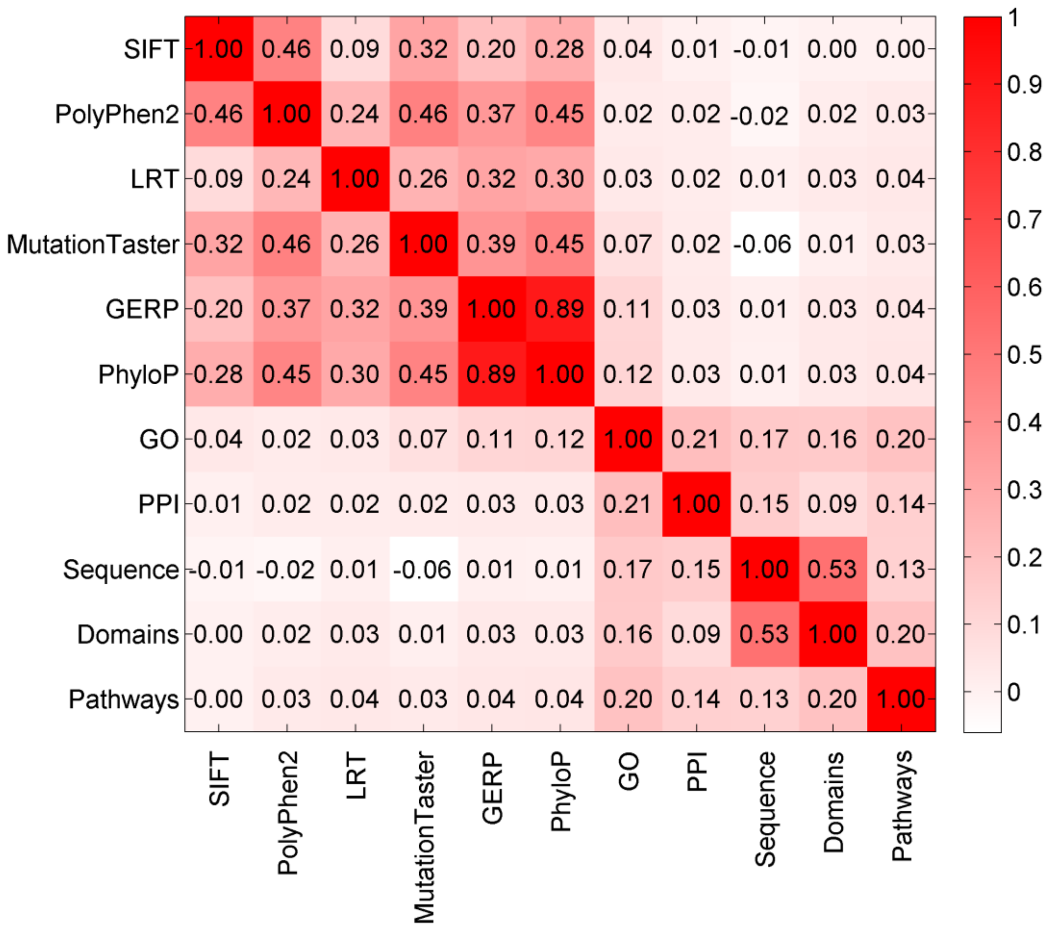

Supplementary Figure 6. Estimated correlations between the 11 data sources used in our studies.

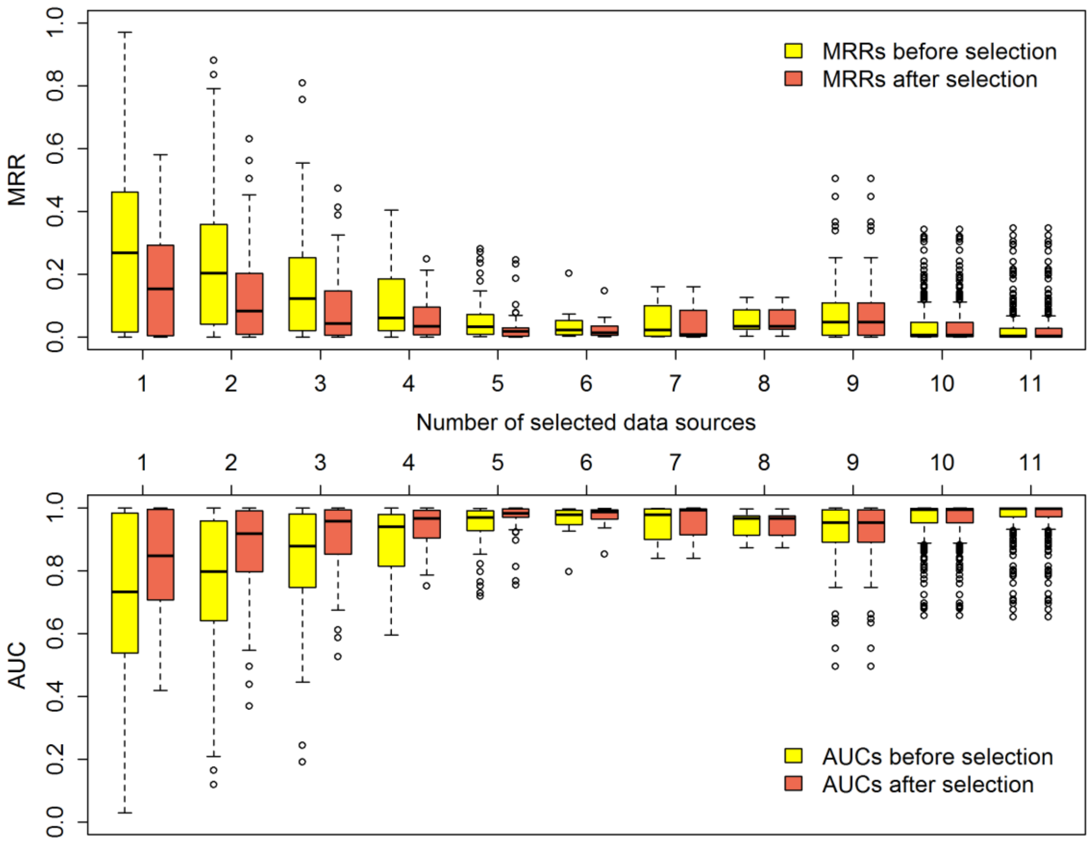

Supplementary Figure 7. Boxplots of MRRs and AUCs before and after the selection of data sources.

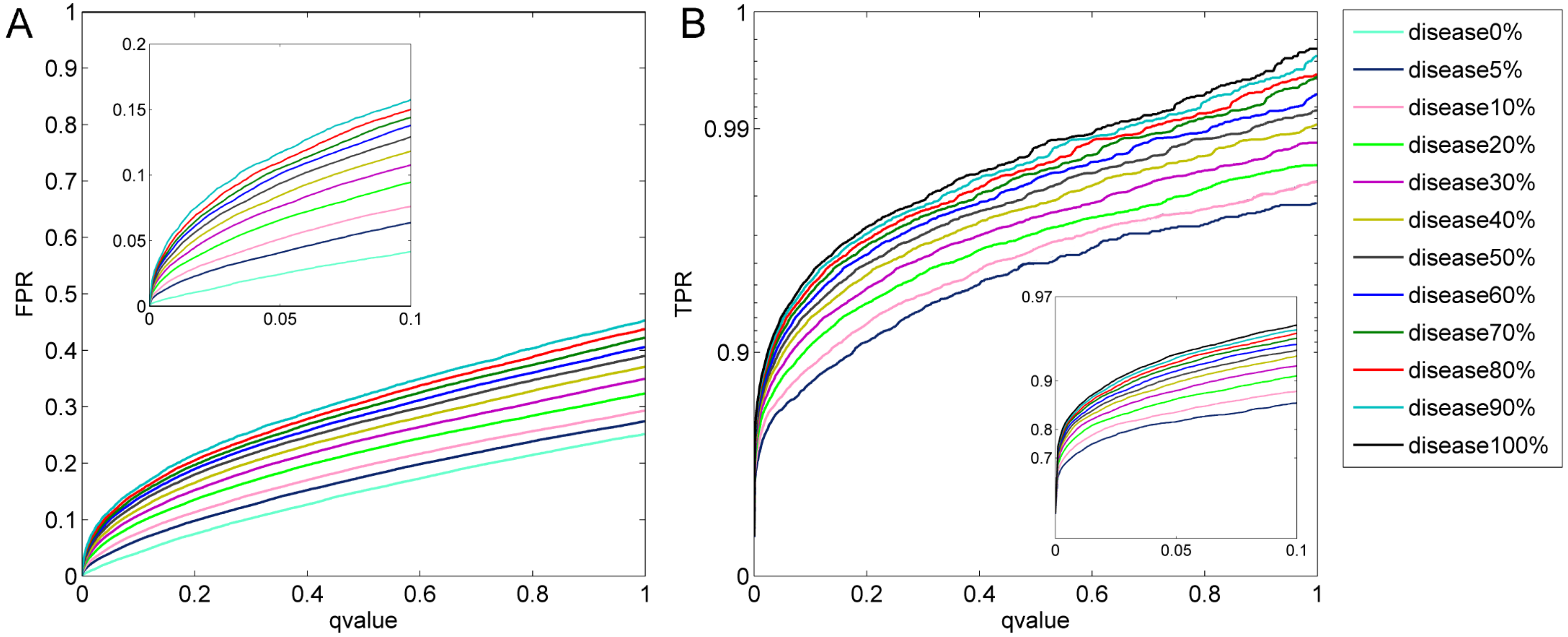

Supplementary Figure 8. Estimated false positive rate (A) and true positive rate (B) under different *q*-value cut-offs.

Supplementary Tables

Supplementary Table 1. Information of the eight HapMap individuals.

Individual	Population	Mapped SNVs	Mapped genes
NA12156	Europe	6766	4145
NA12878	Europe	6711	4160
NA18555	Chinese	6691	4199
NA18956	Japanese	6715	4212
NA18507	Yoruba	8208	4838
NA18517	Yoruba	8180	4831
NA19129	Yoruba	8093	4833
NA19240	Yoruba	8068	4876

Supplementary Table 2. Frequency of occurrence of SNVs in the eight exomes. Group: an SNV is classified into one of the 8 groups according to the number of individuals having the SNV. Number of SNVs in a group: The number of SNVs belonging to a group. Percentage: The number of SNVs in a group divided by 21,947.

**Group**	**1**	**2**	**3**	**4**	**5**	**6**	**7**	**8**	**Total**
**Number of SNVs**	10,739	3,266	1,874	1,436	1,074	1,044	951	1,563	21,947
**Percentage (%)**	48.93	14.88	8.54	6.54	4.89	4.76	4.33	7.12	100

Supplementary Table 3. Jaccard coefficients of SNV sets derived from the eight HapMap individuals.

	NA12156	NA12878	NA18555	NA18956	NA18507	NA18517	NA19129	NA19240
**NA12156**	1	0.4333	0.386	0.3869	0.3147	0.3195	0.3197	0.3165
**NA12878**	0.4333	1	0.3952	0.3972	0.3111	0.3121	0.3212	0.3174
**NA18555**	0.386	0.3952	1	0.4671	0.3177	0.3188	0.3172	0.3198
**NA18956**	0.3869	0.3972	0.4671	1	0.3231	0.3202	0.325	0.3223
**NA18507**	0.3147	0.3111	0.3177	0.3231	1	0.3741	0.3776	0.3759
**NA18517**	0.3195	0.3121	0.3188	0.3202	0.3741	1	0.3757	0.3791
**NA19129**	0.3197	0.3212	0.3172	0.325	0.3776	0.3757	1	0.3814
**NA19240**	0.3165	0.3174	0.3198	0.3223	0.3759	0.3791	0.3814	1

Supplementary Table 4. Data sources selected by the sequential backward selection (SBS) strategy. Original: the data source is present in how many diseases before the selection procedure. Selected: the data source is selected by how many diseases by the SBS strategy. Ratio: Selected / Original × 100%.

Data source	All diseases			Mendelian diseases			Complex diseases			Immune diseases			Neurological disorders		
	Original	Selected	Ratio(%)	Original	Selected	Ratio(%)	Original	Selected	Ratio(%)	Original	Selected	Ratio(%)	Original	Selected	Ratio(%)
SIFT	1434	828	57.74	1376	800	58.14	58	28	48.28	48	31	64.58	263	151	57.41
PolyPhen2	1436	725	50.49	1378	703	51.02	58	22	37.93	48	28	58.33	263	130	49.43
LRT	1429	850	59.48	1371	824	60.10	58	26	44.83	47	30	63.83	262	158	60.31
MutationTaster	1431	723	50.52	1373	701	51.06	58	22	37.93	48	24	50.00	263	138	52.47
GERP	1436	713	49.65	1378	691	50.15	58	22	37.93	48	24	50.00	263	133	50.57
PhyloP	1436	710	49.44	1378	689	50.00	58	21	36.21	48	29	60.42	263	132	50.19
GO	1417	1017	71.77	1360	975	71.69	57	42	73.68	48	30	62.50	260	189	72.69
PPI	1274	933	73.23	1223	893	73.02	51	40	78.43	43	33	76.74	241	178	73.86
Sequence	1432	709	49.51	1375	689	50.11	57	20	35.09	48	26	54.17	263	130	49.43
Domains	1103	596	54.03	1067	580	54.36	36	16	44.44	36	21	58.33	191	109	57.07
Pathways	865	619	71.56	824	587	71.24	41	32	78.05	36	28	77.78	170	124	72.94
